# Receptor Tyrosine Kinases as Therapeutic Targets in Rhabdomyosarcoma

**DOI:** 10.1155/2011/756982

**Published:** 2011-01-02

**Authors:** Lisa E. S. Crose, Corinne M. Linardic

**Affiliations:** ^1^Department of Pediatrics, Duke University Medical Center, Durham, NC 27710, USA; ^2^Department of Pharmacology and Cancer Biology, Duke University Medical Center, Durham, NC 27710, USA

## Abstract

Rhabdomyosarcomas (RMSs) are the most common soft tissue sarcomas of childhood and adolescence. To date, there are no effective treatments that target the genetic abnormalities in RMS, and current treatment options for high-risk groups are not adequate. Over the past two decades, research into the molecular mechanisms of RMS has identified key genes and signaling pathways involved in disease pathogenesis. In these studies, members of the receptor tyrosine kinase (RTK) family of cell surface receptors have been characterized as druggable targets for RMS. Through small molecule inhibitors, ligand-neutralizing agents, and monoclonal receptor-blocking antibodies, RTK activity can be manipulated to block oncogenic properties associated with RMS. Herein, we review the members of the RTK family that are implicated in RMS tumorigenesis and discuss both the problems and promise of targeting RTKs in RMS.

## 1. Introduction

The most common soft tissue sarcomas of childhood and adolescence are rhabdomyosarcomas (RMSs). These malignancies express skeletal muscle markers but are believed to be the result of dysregulated skeletal muscle differentiation of mesenchymal precursors. Like other sarcomas, RMS tumors are molecularly diverse; histological classification separates RMS into two major types, embryonal (eRMS) and alveolar rhabdomyosarcoma (aRMS). As the name implies, eRMS tumors consist of cells morphologically similar to embryonic muscle precursors. The histology of aRMS tumors is distinctive, with clusters of primitive, round cells and open spaces between cell sheets developing upon fixation in formalin, vaguely resembling lung alveoli [1]. The eRMS and aRMS subtypes differ not only in histological appearance but also in prognosis. Patients with eRMS have a generally favorable prognosis, while patients with aRMS do significantly worse, with a five-year survival rate of less than 50% [2]. Furthermore, aRMS can be specified by the presence of a chromosomal translocation resulting in a *PAX3-FOXO1 *(or the less frequent *PAX7-FOXO1, PAX3-NCOA1*, or *PAX3-NCOA2 *[3]) gene product. When metastatic, *PAX3-FOXO1*-positive aRMS patients survive in fewer than 10% of cases [4]. Although staging of RMS still utilizes histology, recent gene profiling studies have suggested that a more accurate classification of RMS might be as fusion gene positive or negative [5, 6]. Thus, modified classification of RMS may lead to better risk stratification at diagnosis and direct appropriate therapy. 

Treatment for RMS has depended on a multimodal approach of surgery, chemotherapy, and radiation. This team strategy has resulted in an overall survival of RMS at about 70% [7]. But as described above, high-risk patients have a poor prognosis, and treatment options are limited. It is believed that without targeted therapies specific for genetic abnormalities associated with RMS, the survival rate will not improve. 

Over the past two decades, research into the molecular mechanisms of RMS has identified key genes and signaling pathways involved in disease pathogenesis. Opportunely, many groups have identified favorable molecular targets for inhibition, such as cell surface receptors. In this review, we will describe the receptor tyrosine kinases (RTKs) associated with RMS and subsequently discuss the therapeutic potential of these targets.

## 2. Receptor Tyrosine Kinases Associated with Rhabdomyosarcoma

### 2.1. IGF-1R

The IGF-1R is a 150-kDa transmembrane RTK expressed on almost all mammalian cells. It is a classical RTK, signaling through ligand occupancy, dimerization, and transmembrane signaling to the cytoplasm through the IRS-1 and IRS-2 adaptor proteins. Present during both embryogenesis and postnatally, IGF-1R is critical for the growth of a variety of mammalian tissue types [8]. In myogenesis, IGF-1R is essential for myoblast proliferation, and IGF ligands induce a strong proliferative response in myogenic precursors. IGF-1R signaling is also necessary for myogenic differentiation through upregulation of the myogenic cascade [9]. There are two known IGF-1R ligands, IGF-1 and IGF-2. While both of these ligands have a ubiquitous tissue distribution, IGF-1 is considered to exert its effects postnatally, while IGF-2 is thought to be dominant during embryogenesis [10]. Through numerous *in vitro* and *in vivo* studies performed by many groups, it is well established that IGF activation of IGF-1R is critical for both proliferation and differentiation of muscle cells. 

The original evidence for upregulation of IGF-1R signaling in RMS came from early studies of IGF ligands in pediatric tumors. As such, IGF-2 was found to be upregulated in both primary RMS tumor samples and cell lines [11, 12], mechanistically the result of loss of imprinting of the maternal or duplication of the active *IGF2* allele [13, 14]. IGF-1R was later found to be upregulated in aRMS by the *PAX3-FOXO1* fusion gene [15]. In this way, increased expression of both IGF-2 and IGF-1R leads to a strong mitogenic feed-forward signaling loop within the tumor.

The role of the IGF-1R signaling pathway in RMS has been examined through experimental loss of function using multiple approaches. Antisense constructs, small molecule inhibitors, and receptor blocking antibodies to IGF-1R have all shown antiproliferative effects in preclinical studies of RMS cell lines and xenografts [12, 16–25]. The mechanism of action appears to be through inhibition of cell proliferation by arrest in the G1 stage of the cell cycle due to downregulation of CDK1 [19, 21]. Interestingly, cell lines that were the most sensitive to IGF-1R blockade were those with the highest levels of IGF-1R expression [16]. 

**Figure 1:**
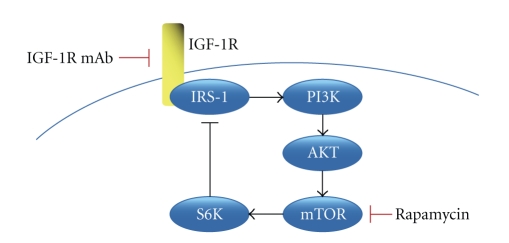
Rationale for dual treatment targeting the IGF-1R signaling pathway in RMS. Rapamycin inhibits mTOR signaling, preventing inhibitory feedback on IRS-1 which allows proliferative signals from IGF-1R to IRS-1, PI3K, and AKT. Dual treatment using rapamycin in combination with IGF-1R inhibition, such as monoclonal blocking antibodies, prevents signaling to these critical progrowth signaling nodes.

An understanding of the signaling pathways downstream of IGF-1R has been enhanced through studies using the small molecule inhibitor, rapamycin. Rapamycin inhibits mTOR, a PIKK family member kinase that responds to changes in nutrient availability and cellular stresses. RMS sensitivity to rapamycin is mediated by IGF-1R signaling, demonstrating that the mTOR pathway is downstream of IGF-1R [17, 26]. As shown in Figure 1, in the IGF-1R signaling pathway, IGF-1R signals to IRS-1 and AKT, which then signals to mTOR. Paradoxically, treatment of cancer cells with rapamycin activates AKT, due to blockade of a feedback loop via ribosomal S6 kinase (S6K) that normally inhibits IRS-1 [27]. This effect can be reversed by inhibiting IGF-1R. Through dual treatment of RMS tumors with rapamycin and IGF-1R inhibitors, the proliferative IGF-1R signaling cascade can be dramatically reduced. In this way, IGF-1R blockade has become an attractive proposed treatment for RMS and other IGF-driven cancers [16, 28, 29]. 

IGF-1R inhibitors are one of many classes of compounds tested in the Pediatric Preclinical Testing Program (PPTP). This NCI-funded program provides a preclinical screening platform to test new agents that may have activity against pediatric cancers. As shown in Table 1, the IGF-1R inhibitor IMC-A12 showed effectiveness in RMS xenografts, while SCH 717454 had a partial effect. These studies, in addition to the preclinical data described above, provide a strong rationale to pursue IGF-1R inhibitors in clinical trials for pediatric RMS patients. 

As shown in Table 2, phase I and phase II trials of RTK small molecule inhibitors, monoclonal antibodies against RTK ligands, and monoclonal antibodies against RTKs have been under investigation since the year 2000. Notably, inhibition of IGF-1R with monoclonal antibodies has been the most recent focus of these trials and, if successful, will be the first FDA-approved RTK-targeted therapy for RMS.

### 2.2. MET

MET is a proto-oncogene RTK necessary for cell proliferation, motility, and epithelial-mesenchymal transition. Similar to IGF-1R, MET is also 150 kDa and shows broad tissue expression in embryonic and postnatal tissues. In contrast to IGF1-1R, MET has only one ligand, termed hepatocyte growth factor (HGF). In the context of myogenesis, limb mesenchyme secretes HGF, which directs myogenic precursors to the limb bud. In this way, MET signaling regulates delamination and migration of muscle precursors from the embryonic dermomyotome [30]. MET also promotes cell proliferation in muscle precursors when activated with HGF* in vitro* [31]. When these cells stop proliferating and induce differentiation, HGF and MET expression decreases [32]. Thus, MET promotes both myoblast proliferation and migration processes in normal cells. 

HGF is also known as “scatter factor,” referring to its ability to induce cell motility. Accordingly, MET has been implicated in cytoskeletal reorganization and migration in cancer cells. In RMS cells, HGF promotes chemotaxis and invasion [33–35]. Because of these migratory effects of HGF/MET signaling, the role of this signaling pathway in tumor cell metastasis has been examined. Cells derived from bone marrow secrete HGF, and RMS cells have been shown to home to bone marrow, due in part to MET expression [33, 35, 36]. Furthermore, bone marrow aspirates from RMS patients with metastatic disease have elevated MET expression [37]. Therefore, a major role for MET is to confer migratory and metastatic properties.

MET-null and *Splotch* (*PAX3* mutant) mice both exhibit loss of muscle precursor colonization in the limb bud [30, 38], which revealed an association of PAX3 with MET expression [39]. MET is a transcriptional target of PAX3 and PAX3-FOXO1, and RMS cell lines and tumors express elevated levels of MET compared to normal muscle [34, 40, 41]. Targeted knockdown of MET in human RMS cell lines decreases RMS cell proliferation* in vitro* and tumor burden in mouse xenograft models [35, 42]. Therefore, in addition to regulating migration and metastasis, MET also appears to regulate proliferative properties in RMS. 

Several genetically engineered mouse models of RMS either exploit MET signaling or demonstrate deregulated MET expression. The most robust murine model of RMS was generated through manipulation of the HGF/MET signaling axis. While transgenic *HGF* mice were predisposed to a low incidence of many types of cancers, including skeletal muscle-derived tumors [43], transgenic *HGF* mice with a targeted deletion of the *INK4A/ARF* locus had a near complete penetrance of eRMS in young animals [44]. Mouse models utilizing the *PAX3-FOXO1* fusion gene have also defined roles for MET. Conditional replacement of *PAX3* with *PAX3-FOXO1* results in abnormal delamination of myogenic progenitors from the somite that can be reversed with expression of a kinase-inactive MET [45]. aRMS has been modeled by conditional *PAX3-FOXO1* at the *PAX3* locus in either an *INK4A/ARF* or *p53*-null background. MET upregulation was observed in all tumors derived, regardless of the genetic background [46]. 

Although there is clear evidence for the involvement of MET in RMS initiation, progression, and metastasis, to date there have been no clinical trials evaluating MET inhibition in the context of RMS. Since MET is implicated in many adult malignancies, and phase I clinical trials for monoclonal antibodies and small molecule inhibitors with anti-MET activity have recently begun, we should expect to see trials recruiting pediatric RMS patients.

### 2.3. EGFR, ErbB2

The ErbB family of RTKs is comprised of four members: EGFR (ErbB1, HER1), ErbB2 (HER2), ErbB3 (HER3), and ErbB4 (HER4). Members of this family are similar in size, at 190 kDa, and each are necessary for embryonic development. Each has been implicated in cancer initiation and progression but in different tissue types. Notably, ErbB3 is a noncatalytic receptor but exerts an oncogenic function through heterodimerization with other ErbB family members [47]. ErbB receptors regulate multiple levels of cell physiology in different tissues, including cytoskeletal rearrangement, proliferation, and evasion of apoptosis. In mouse myoblasts, EGFR is expressed and is active in both undifferentiated and differentiated cells. EGFR blockade in murine myotubes induced cell death, suggesting that EGFR regulates prosurvival signaling in myogenic cells [48]. EGFR has been associated with many adult malignancies, including breast, non-small cell lung cancer, glioblastoma, head and neck, gastric, genitourinary, and colorectal carcinomas [49]. Prognosis in these cancers can often be estimated by the presence or absence of EGFR mutations, deletions, or overexpression. 

ErbB family proteins were found to be expressed in RMS cells during screening for growth factor signaling pathway members [51–53]. While EGFR is more highly expressed in eRMS tumor tissue [54–56] ErbB2 expression is more prevalent in aRMS tumor tissue, and found in the majority of RMS tumors in the head and neck [55, 57]. ErbB3 is also expressed in RMS cells and may play a role in regulating differentiation, but ErbB4 has not been found to be expressed in RMS cells [52]. Notably, to date no mutations have been identified in the ErbB genes in RMS. Blocking EGFR expression by antisense methods decreases RMS cell proliferation *in vitro* [58]. Unfortunately, follow-up preclinical testing of EGFR inhibitors *in vivo* has not shown efficacy. As an example, the small molecule inhibitor lapatinib was tested in a PPTP screen but had little effect in solid tumors, suggesting that EGFR inhibition alone is not sufficient to inhibit tumorigenesis. 

Expression of an activating ErbB2 mutation in combination with loss of p53 is sufficient to induce rhabdomyosarcoma in mouse models. The resulting tumors appear histologically similar to eRMS and express IGF-2 and IGF-1R [59]. This model was used to test a cancer vaccine developed against the ErbB2 receptor, which was successful in preventing spontaneous RMS formation in 50% of mice examined [60]. Even though preclinical studies have not shown promise as monotherapy, ErbB2 may play a supportive role in RMS initiation. 

 Although inhibition of a single RTK may be beneficial in some circumstances, studies have suggested that this approach will likely not be sufficient treatment for RMS, and this appears to be true of EGFR. This has been appreciated in preclinical models, and therefore human clinical trial design has been modified to evaluate RTK inhibition in combination with a cytotoxic agent. Gefitinib, a small molecule inhibitor for EGFR, is being tested in phase I clinical trials in pediatric solid tumors, in combination with irinotecan [61]. Phase I clinical trials have been completed for erlotinib, which also targets EGFR, done in combination with temozolomide with few adverse effects [62].

### 2.4. PDGFR

The PDGFR family of RTKs includes PDGFR*α* and PDGFR*β*, both 200 kDa in size, which homo- or heterodimerize to perform their signaling functions. While PDGFR*α* is believed to be critical in the development of neural, epithelial, and skeletal tissues, PDGFR*β* is important for blood vessel formation and hematopoiesis [63]. In normal myogenesis, PDGFR activation is downregulated, implying that loss of PDGFR signaling is involved in the cell cycle exit that accompanies differentiation [64]. There are four ligands for PDGFR, PDGF-A through PDGF-D. In myoblasts, PDGF-B promotes cell migration and proliferation and reduces differentiation* in vitro *[65, 66]. Therefore, PDGFR signaling is important for embryogenesis, and specifically myogenesis by regulating proliferation, migration, and differentiation in myogenic precursors. 

In RMS, the two PDGF receptors show increased expression [67–69], and PAX3-FOXO1 has been shown to activate transcription of PDGFR*α* [70]. Imatinib, a small molecule inhibitor of PDGFRs, has shown promise as an RMS therapy in preclinical models. In a mouse genetic model of RMS, high expression of PDGFR*α* was observed in advanced tumors. Loss of function of PDGFR*α* through siRNA or imatinib induced tumor cell apoptosis. When imatinib or PDGFR*α* blocking antibodies were used to treat RMS tumors in these mice, 50% of mice had at least a partial reduction of tumor growth [71]. PDGFR inhibition with sunitinib or sorafenib showed promise in PPTP screening, promoting tumor growth delay or inhibition, respectively (Table 1). However, both sunitinib and sorafenib are known to inhibit numerous other kinases, so these effects may not be due to PDGFR inhibition alone. Although targeting PDGFR alone has shown some promise in preclinical models, combination treatment with other chemotherapies may be more beneficial. In a mouse xenograft model of RMS, significant reduction of tumor burden was observed when imatinib was used in combination with the topoisomerase inhibitor topotecan [68]. 

In RMS patients, high expression of PDGFRs is associated with decreases in failure-free and overall survival, implicating PDGFR signaling in advanced stages of the disease [53, 72]. The PDGFR inhibitor imatinib was tested in advanced sarcomas of various types in a phase II trial. Overall, the results did not support the use of imatinib as a monotherapy, as only 2% of participants saw an effect resulting in partial or complete remission [73]. However, combination treatment of imatinib with other targeted or cytotoxic agents may be more beneficial, as was seen in preclinical models [68].

### 2.5. VEGFR

The VEGFR family of RTKs is comprised of three members: VEGFR1 (FLT1), VEGFR2 (FLK1/KDR), and VEGFR3 (FLT4). VEGFRs on endothelial cells regulate multiple levels of angiogenesis by promoting endothelial cell proliferation, migration, sprouting, and survival. These receptors are activated by the five members of the VEGF ligand family, VEGF-A through VEGF-D, and placental growth factor (PlGF). VEGFs are produced in a wide variety of tissues in response to hypoxia, in order to recruit vasculature to the hypoxic area [74]. Interestingly, VEGFRs are also expressed in myoblasts, and VEGF promotes myoblast migration and survival. VEGFR expression is downregulated upon myogenic differentiation, suggesting that prolonged VEGFR signaling negatively regulates differentiation [75]. 

Similar to other cancer cells, when exposed to hypoxia, RMS cells increase their secretion of VEGF [28]. RMS cells express multiple isoforms of VEGF and VEGFRs, implying that RMS tumors may utilize an autocrine loop to not only promote tumor vascularity but induce tumor growth as well. This is supported by evidence that treatment of RMS cells in culture promotes proliferation, while treatment with VEGFR antibody blocked this effect [76]. Furthermore, inhibition of signaling downstream of VEGFR prevents expression of VEGF by RMS cells, suggesting a feed-forward loop promoting proliferation [77]. 

VEGFR inhibitors have shown promising results in preclinical studies. Monoclonal antibodies to VEGF and VEGFRs and small molecule inhibitors to VEGFRs have been tested in mouse xenografts of RMS reduced tumor volume and vascularity [50, 78–81]. Notably, cisplatin-resistant RMS cells have increased expression of VEGF and VEGFRs, implicating this autocrine signaling in RMS cell survival. Cisplatin-resistant cells were sensitive to VEGFR inhibition, which also blocked VEGF expression [82]. In this way, highly aggressive tumor cells could be targeted with anti-VEGFR therapy. In PPTP screening, VEGFR inhibitors have also shown potential. The inhibitors AZD2171, sorafenib, and sunitinib have each inhibited tumor growth in RMS xenograft models. Currently, there are multiple clinical trials testing VEGFR inhibition in RMS patients. These include the small molecule inhibitors sunitinib, pazopanib, and cediranib as well as the VEGF monoclonal antibody bevacizumab. These studies are ongoing, but whether the strong preclinical data for VEGFR inhibition will translate to positive outcomes in clinical trials remains to be seen.

### 2.6. FGFR

The FGF receptor (FGFR) family consists of four members, FGFR1 through 4, and vary in size (120–160 kDa), tissue distribution, and ligand affinity. FGFRs affect many aspects of cell and organism physiology including proliferation, migration, and differentiation through activation by FGF ligands, of which there are at least 23 [83]. FGFR4 is considered to be the predominant FGFR in skeletal muscle, regulating skeletal muscle differentiation in chick models and muscle regeneration in mice [84, 85]. As is true for most of the RTKs reviewed here, FGFR4 expression in myoblasts decreases during differentiation, implying that FGFR4 is important in myogenic precursors [86]. 

While FGFR1 and FGFR3 have been observed to have increased expression in isolated RMS cases [87, 88], and FGF ligands are expressed in RMS cells and tissues [51, 89], signaling through FGFR4 has been the best characterized in RMS tumorigenesis. FGFR4 expression is upregulated in RMS cell lines and tumors [90, 91], and PAX3-FOXO1 promotes FGFR4 expression through 3′ enhancer regions [15]. Recently, FGFR4 was characterized as a regulator of RMS tumor growth and metastasis. Activating mutations within the kinase domain of FGFR4 were identified in 7% (7 of 94) of RMS cases, demonstrating overactive FGFR4 signaling in RMS. These activating mutations were sufficient to transform cells, increase RMS lung metastasis, and decrease survival in mouse xenograft models. Blocking FGFR4 expression decreased RMS tumor size, cell migration, and metastasis, therefore characterizing FGFR4 as a possible molecular target for RMS [92]. FGFR4 is the most recent RTK implicated in RMS, and as such more research will be needed to verify that FGFR4 is a rational therapeutic target to pursue in preclinical and clinical settings.

## 3. Regulation of RTK Expression in Rhabdomyosarcoma

Since a signature mutation of aRMS is a chromosomal translocation resulting in the PAX3/7-FOXO1 or PAX3-NCOA1/2 transcription factors, much attention has been focused on the genes regulated by these fusion proteins. In fact, IGF-1R, MET, PDGFR, VEGFR1, and FGFR4 have all been shown to be regulated by PAX3-FOXO1 either by activation of the RTK gene promoter or through 5′ or 3′ enhancing elements [15, 39, 70, 93]. Since transcription factors are generally difficult to target due to chemical intractability, druggable PAX3-FOXO1 transcriptional targets such as RTKs could be more promising than inhibiting PAX3-FOXO1 itself. 

Another regulator of RTKs in RMS is the tumor suppressor p53. The importance of p53 function in RMS has been underscored by its role in promoting RMS in mouse models when it is absent [46, 59]. Mutations in p53 have been documented in both histological subtypes of RMS [94], and IGF-1R and PDGFR have been definitively shown to be upregulated in p53 loss-of-function experiments and downregulated when nonmutated p53 is added back to these systems [71, 95]. Although PAX3-FOXO1 and p53 regulation of RTK transcription in specific cases has been informative, there is a need for a better understanding of when and how RTK transcription is activated in RMS tumorigenesis. 

Most recently, posttranscriptional regulation of genes has been shown to play a role in RMS tumorigenesis. The most focus has been on microRNAs mir-1 and mir-206, so-called “myo-mirs.” Upon myogenic pathway induction, mir-1 and mir-206 expression is upregulated, leading to post-transcriptional downregulation of mir-1 and mir-206 targets. Mir-1 and mir-206 have been found to be sufficient to induce myogenic differentiation in myoblasts [96]. RMS cell lines and tumors do not express mir-1 and mir-206 and therefore are not able to posttranscriptionally regulate mir-1 and mir-206 targets. Surprisingly, MET was found to be implicated in the mir-206 pathway. MET contains two putative binding sites for mir-206 in the MET 3′ untranslated region. Ectopic expression of mir-206 caused loss of MET expression, induction of skeletal muscle differentiation markers, loss of cell proliferation, and decreased tumor burden in mouse RMS xenografts [97, 98]. The mechanism behind the loss of mir-1 and mir-206 in RMS remains to be determined, but its potential to downregulate therapeutic targets like MET may hold promise for RMS treatments in the future.

## 4. Therapeutic Potential for RTK Inhibition in Rhabdomyosarcoma

As druggable receptors at the plasma membrane, RTKs have been the focus of intense basic and pharmacologic research. Small molecule inhibitors, ligand-neutralizing agents, and monoclonal receptor-blocking antibodies have been generated for many of the RTKs expressed in RMS. However, it is not likely that all of the RTKs in RMS will survive the tests of robust preclinical testing and be evaluated in clinical trials. Therefore, determining which target(s) are the most promising and worthy of clinical trial assignment will be critical. As described below, understanding their mechanisms of upregulation, acquired resistance, and pathway crosstalk will be key to determining how to pharmacologically exploit RTK signaling in RMS. 

RTKs are only one of numerous and diverse signaling pathways upregulated in cancer, so identifying the RTKs upregulated in RMS is only a starting point to determine their potential as therapeutic targets. In many cases, blockade of an upregulated RTK will cause cytostatic growth inhibition but not eliminate the cancer cells completely, leading to emergence of resistant clones and refractory disease. However, if the cells have become dependent on a particular RTK signaling pathway for survival, so-called “oncogene addiction” [99], blockade of these pathways should be more effective in disease eradication. The challenge then becomes determining which RTKs confer oncogene addiction. 

Another possibility is the presence of activating mutations in RTKs. RTKs containing an activating mutation are much more sensitive to inhibitors targeting that RTK than cells or tumors with a wild-type RTK. An example of this phenomenon was observed in non-small cell lung carcinoma. In clinical trials of EGFR inhibitors, only 10% of patients responded to treatment. Upon further investigation, it was found that the responding patients harbored somatic, activating EGFR mutations [100]. Similarly, in PPTP screening, Kasumi-1 cells (which contain an activating c-KIT mutation) were found to be particularly sensitive to sorafenib [101]. To date, FGFR4 is the only RTK known to have an activating mutation in RMS [92]. Deep sequencing of RTKs implicated in RMS will need to be done to address the possibility that other RTKs are mutated in RMS. A second possibility is genomic amplification or deletion or sheer upregulation of RTKs or their signaling components. This has already proven important in our understanding of IGF-1R in RMS, as the loss of imprinting of the *IGF-2* gene, or higher expression levels of IGF-1R, lead to oncogene dependence even in the absence of an activating mutation and confer sensitivity to RTK blockade [13, 16]. Similarly, wild-type EGFR expression is upregulated in RMS cells. Although inhibition of EGFR does not appear to be a promising candidate for monotherapy, recent studies have suggested that EGFR could be used in targeted immunotherapy applications [102]. In sum, understanding the underlying genetic changes as well as utilizing upregulation of RTKs through novel treatments will guide future RMS therapies.

A drawback of targeted therapies is the ability of tumor cells to adapt and acquire resistance. Through further upregulation of the therapeutic target, mutation of the therapeutic target, or upregulation of a compensating RTK or signaling pathway, cancer cells can rapidly adjust to promote tumor cell survival [103]. For example, in the case of IGF-1R blockade, RMS cells resistant to IGF-1R small molecule inhibitors were found to have increased expression of EGFR when compared to those cells that were sensitive. To this end, dual treatment with IGF-1R and EGFR inhibition increased the antitumor effect in RMS mouse xenograft models [24]. Understanding how and if RMS cells adapt to targeted therapies will be critical for successful treatment options. 

Although induction of resistance may pose a problem for targeted therapy in RMS, crosstalk within signaling pathways could provide a way to exploit RTK inhibition. For example, the IGF-1R and VEGFR pathways exhibit crosstalk in RMS, and by experimentally inhibiting IGF-1R signaling, VEGF secretion is reduced [28]. Thus, IGF-1R blockade has the potential to thwart both IGF-1R and VEGFR pathways. In addition, many RTKs utilize redundant downstream signaling components. Targeting more than one RTK through multiple individual inhibitors, or using a less specific inhibitor to block several RTKs simultaneously, may prove beneficial by strong inhibition of signaling at a common node. Through a systematic and comprehensive analysis of other as yet undescribed crosstalk mechanisms in RMS, these dependencies can be identified and provide the basis for further preclinical testing.

**Figure 2:**
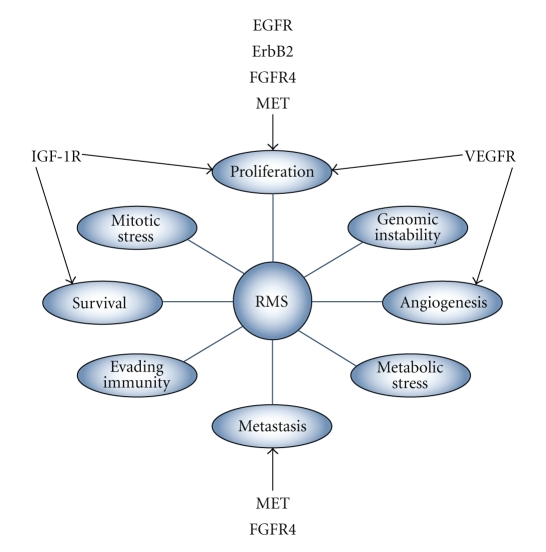
RTKs associated with RMS and their known roles in RMS tumorigenesis or progression.

In addition to the theoretical attraction of combination therapy on blocking signaling pathways, dual or multiple inhibition of RTKs may offer beneficial effects in inhibiting the unique tumorigenic properties of cancer cells. The “hallmarks of cancer” as defined by Hanahan and Weinberg [104], and further classified by Negrini et al. [105], represent specific tumorigenic properties of the cancer cell, as shown in Figure 2. The RTKs we have described impact various cancer cell characteristics. Through targeting of individual RTKs, therapeutic intervention could inhibit distinct malignant properties. When blocked in combination, inhibition of multiple RTKs could have a profound effect on tumor growth and progression. Realistically, these positive outcomes on tumor inhibition may be offset by increased incidence of toxicity and side effects. Therefore, until both pre-clinical and clinical studies address these issues, combination targeted therapy will pose a sizeable challenge for researchers.

## 5. Prioritization of Therapeutic Targets

One of the most daunting challenges for pediatric oncology clinical trial design is how to identify the strongest therapeutic candidates to pursue. Because of the limited number of pediatric patients, only the most promising agents under development as cancer treatment should be evaluated in a clinical trial setting. As mentioned above, the PPTP provides a preclinical platform to screen an experimental compound against many types of pediatric cancers. To be considered for a PPTP screen, there must be significant rationale for the proposed agent in pediatric cancers, including the mechanism of action and *in vitro* and* in vivo *efficacy. In some cases, evidence from pediatric preclinical models or adult clinical trials is available, providing pharmacokinetic and dosing data. These cases may receive priority, as they expedite some of the issues addressed in early clinical trials. In terms of prioritization of RTK targets, there is a need to understand the genetic foundation behind activation of specific RTK signaling pathways in RMS cells. Genetic screening of RTKs for mutations or analysis of downstream signaling pathways may provide insight into which therapeutic candidates could have the most profound effects on RMS cells. Clearly, both large scale screens and mechanistic validation will be necessary to prioritize the many candidates. 

There are still numerous RTK targets that could be utilized for RMS therapy and warrant further study to provide additional treatment opportunities. Additional study of these targets in preclinical models will be necessary to advance their use to clinical trials, either as single targeting agents, multiple targeting agents, or single targeting agents in combination with cytotoxic therapy. As we expand our knowledge of how RTKs function individually or together, the potential for utilizing RTK inhibition could be a turning point in a new era of targeted therapy for RMS. The future of RMS therapies holds promise and will provide improved options for RMS patients, including those in high risk groups.

## Figures and Tables

**Table 1:** Results of RTK inhibitors used in Pediatric Preclinical Testing Program.

Intended target	Inhibitor	Additional targets	*In vivo *response, RMS xenografts*	Conclusions	Ref.
EGFR, ErbB2	Lapatinib	Erk1/2, Akt	1/5	Limited effectiveness in all xenografts tested	[30]
IGF-1R	IMC-A12	N/A	6/6	Tumor growth inhibition in most solid tumor xenografts, most effective in RMS xenografts	[25]
IGF-1R	SCH 717454	N/A	2/4	Tumor growth inhibition in many solid tumor xenografts	[31]
PDGFR	Sunitinib	c-KIT, VEGFR2, FLT3	5/6	Tumor growth delay, inhibition in most solid tumor xenografts	[32]
Raf1	Sorafenib	VEGFR, PDGFR, RET, FLT3, c-KIT	2/6	Tumor growth inhibition in various tumor xenografts	[33]
SRC	Dasatinib	ABL, c-KIT, EPHA2, PDGFR	1/6	Limited effectiveness in solid tumor xenografts	[34]
VEGFR1-3	AZD2171	PDGFR, c-KIT	5/5	Tumor growth inhibition in most solid tumor xenografts	[35]

*Xenografts with “intermediate” or “high” response activity as defined by Maris et al. [50].

**Table 2:** Clinical trials evaluating drugs that target RTKs or their ligands, with strata that include rhabdomyosarcoma.

RMS tumor eligibility	Patient age (years)	Drug	Intended RMS Target	Additional Targets	Phase	Start date	Sponsor/ collaborator
Small molecule inhibitors							

Relapsed/refractory	≥15	Imatinib	PDGFR	ABL, c-Kit	I/II	Aug 2000	EORTC
Resistant	≥15 but ≤70	Imatinib	PDGFR	ABL, c-Kit	II	Feb 2001	Novartis
Advanced	≥10	Imatinib	PDGFR	ABL, c-Kit	II	Jun 2002	NCI
Refractory	≤21	Erlotinib	EGFR		I	Feb 2004	COG/NCI
Refractory	≤21	Gefitinib	EGFR		I	Sep 2005	St. Jude's/Astra Zeneca
Metastatic/advanced/ recurrent	≥18	Sunitinib	PDGFR	c-KIT, VEGFR2, FLT3	II	Apr 2007	MSKCC/NCI
Advanced	≥13	Dasatinib	SRC	ABL, c-KIT, EPHA2, PDGFR	II	May 2007	SARC/ Bristol-Myers Squibb
Metastatic/recurrent	≥1 but ≤25	Dasatinib	SRC	ABL, c-KIT, EPHA2, PDGFR	I/II	Sep 2008	Beckman Research Institute/ NCI
Metastatic/relapsed/ refractory	≥18	Pazopanib	VEGFR1-3	PDGFR, c-KIT	III	Oct 2008	EORTC
Refractory/recurrent	≥2 but ≤18	Cediranib	VEGFR1-3		I	Dec 2008	NCI

Monoclonal antibodies against RTK ligands							

Metastatic	≥0.5 but ≤18	Bevacizumab	VEGF	N/A	II	Jul 2008	Hoffman-La Roche

Monoclonal antibodies against RTKs							

Recurrent/refractory	≥2	R1507	IGF-1R	N/A	II	Nov2007	Hoffmann-La Roche/SARC
Unresectable/locally advanced/ metastatic	≥16	IMC-A12	IGF-1R	N/A	I/II	Jun 2008	U. Chicago/NCI
Metastatic/advanced	≥12	IMC-A12	IGF-1R	N/A	II	Jun 2008	ImClone LLC
Relapsed/refractory	≤30	Cixutumumab	IGF-1R	N/A	II	Jan 2009	COG/NCI
Metastatic	≤49	Cixutumumab	IGF-1R	N/A	Pilot	Jan 2010	COG/NCI

Obtained from clinicaltrials.gov website September 2010.
